# X-ray diffractometry for the structure determination of a submicrometre single powder grain

**DOI:** 10.1107/S090904950900675X

**Published:** 2009-04-01

**Authors:** Nobuhiro Yasuda, Haruno Murayama, Yoshimitsu Fukuyama, Jungeun Kim, Shigeru Kimura, Koshiro Toriumi, Yoshihito Tanaka, Yutaka Moritomo, Yoshihiro Kuroiwa, Kenichi Kato, Hitoshi Tanaka, Masaki Takata

**Affiliations:** a JASRI/SPring-8, 1-1-1 Kouto, Sayo-cho, Sayo-gun, Hyogo 679-5198, Japan; b Japan Science and Technology Agency, CREST, 5 Sanbancho, Chiyoda-ku, Tokyo 102-0075, Japan; c Department of Applied Chemistry, Faculty of Science and Engineering, Chuo University, 1-13-27 Kasuga, Bunkyo-ku, Tokyo 112-8551, Japan; d Graduate School of Material Science, University of Hyogo, 3-2-1 Kouto, Kamigori-cho, Ako-gun, Hyogo 678-1297, Japan; e RIKEN SPring-8 Center, Harima Institute, 1-1-1 Kouto, Sayo-cho, Sayo-gun, Hyogo 679-5148, Japan; f The Graduate School of Pure and Applied Sciences, University of Tsukuba, Tenodai, Tsukuba, Ibaraki 305-8571, Japan; g Department of Physical Science, Graduate School of Science, Hiroshima University, 1-3-1 Kagamiyama, Higashi-Hiroshima, Hiroshima 739-8526, Japan; h Department of Advanced Materials Science, School of Frontier Sciences, The University of Tokyo, 5-1-5 Kashiwanoha, Kashiwa-shi, Chiba 277-8561, Japan

**Keywords:** submicrometre X-ray beam, phase zone plate, X-ray diffraction, single-crystal structure analysis, powder diffraction

## Abstract

A high-precision diffractometer has been developed for the structure analysis of a submicrometre-scale single grain of a powder sample at the SPring-8 BL40XU undulator beamline. The key design concept is the combination of a stable focused synchrotron radiation beam and the precise axis control of the diffractometer, which allows accurate diffraction intensity data of a submicrometre-scale single powder grain to be measured. The phase zone plate was designed to create a high-flux focused synchrotron radiation beam. A low-eccentric goniometer and high-precision sample positioning stages were adopted to ensure the alignment of a micrometre-scale focused synchrotron radiation beam onto the submicrometre-scale single powder grain. In order to verify the diffractometer performance, the diffraction pattern data of several powder grains of BaTiO_3_, of dimensions ∼600 × 600 × 300 nm, were measured. By identifying the diffraction data set of one single powder grain, the crystal structure was successfully determined with a reliable factor of 5.24%.

## Abstract 2

A high-precision diffractometer with a synchrotron radiation microfocusing technique has been developed to investigate the crystal structure of a submicrometre-scale single grain of powder sample. The structure of a BaTiO_3_ single powder grain, of dimensions ∼600 × 600 × 300 nm, was determined.

## 1. Introduction

Up to now, the structure identification and characterization of various new materials have been carried out by powder diffraction experiments as newly synthesized materials are, in most cases, obtained in powder and/or in polycrystalline forms. This research technical trend has been accelerated by the high-photon-flux monochromatic X-rays of synchrotron radiation in addition to the progress of computers and software for structure determination. Consequently, the development of an *ab initio* structure analysis technique, which denotes unknown structure determination, is now one of the most topical research subjects in powder crystallography (David *et al.*, 2002 ▶; David & Shankland, 2008 ▶). On the other hand, an X-ray synchrotron radiation beam with high brilliance and small divergence allows an alternative method to be suggested, which can determine the crystal structure of a micrometre- or submicrometre-scale single crystal corresponding to a single grain of a powder sample. In fact, some structure determinations of a few micrometre-sized single crystals were recently reported using a synchrotron radiation focusing technique (Riekel *et al.*, 2005 ▶; Volkringer *et al.*, 2007 ▶). Before those works, the Laue method, which uses an intense white synchrotron radiation beam instead of a focused monochromatic beam, was also performed on a submicrometre crystal to measure the intensity data efficiently (Ohsumi *et al.*, 1991 ▶). However, the conventional structure determination of a submicrometre-scale single crystal has not yet been reported. This is mainly due to three technical difficulties which must be overcome: the accurate intensity data collection of very weak diffraction spots, the precise centring of the invisible sample, and the infallible manipulation of a submicrometre-scale single crystal. The combination of the synchrotron radiation focusing technique and the precise axis control of the diffractometer is a solution for the first and second difficulties. The latest focusing technique for a synchrotron radiation diffraction experiment can firmly produce a micrometre-scale high-flux synchrotron radiation beam. The precise axis control enables the submicrometre-scale sample to be moved and kept within the micrometre-scale focused synchrotron radiation beam. We have therefore developed and optimized a high-precision diffractometer which combines a synchrotron radiation focusing technique and low eccentric sample rotation at the SPring-8 BL40XU undulator beamline, to enable the structure analysis of a submicrometre-scale single powder grain. As for the third difficulty, since the development of the systematic manipulation techniques to select and capture one single grain of powder sample is still in progress, several submicrometre-scale single powder grains have been attached to a fine glass fibre by means of the conventional sample manipulation technique.

From a technical viewpoint, the structure analysis of a submicrometre-scale single powder grain has great advantages for applications to structural materials science research. Firstly, we can remove the intrinsic problem in the powder diffraction method, peak overlapping, which causes ambiguities in the determined structure in most cases, since a single powder grain can be mostly treated as a single crystal. Secondly, submicrometre-scale samples enable us to measure data free from extinction and/or absorption correction.

Another advantage arises in the case of observations of photo-induced phase transition (PIPT). For laser-pump and synchrotron-radiation-probe experiments, we can avoid the mismatch between the penetration depths of laser and X-rays since a typical penetration depth of laser light is of the order of 1 µm. This indicates near 100% yield of PIPT, which greatly improves the accuracy of the structure analysis of PIPT, is achievable with a submicrometre-scale single powder grain.

Practically, the structure analysis of a submicrometre-scale single powder grain can be directly applied to the research of the grain-size-dependent structure–property relationship. One of the most attractive examples is a barium titanate (BaTiO_3_) fine particle, which shows a phase transition from tetragonal to cubic as the particle size decreases. Some diffraction studies of the size effect of BaTiO_3_ were carried out using the powder diffraction method (Aoyagi *et al.*, 2002 ▶). However, the powder diffraction method had the following disadvantages: the obtained structural parameters are the averaged information from various size particles in the powder sample; in addition, the peak overlapping becomes more serious owing to the peak profile broadening caused by the small particle size. The structure analysis of a single powder grain could overcome these difficulties because the diffraction peaks are isolated geometrically.

In this article we illustrate the concepts and features of the high-precision diffractometer for the structure analysis of a single powder grain, with an example of the structure analysis of a BaTiO_3_ fine particle of dimensions ∼600 × 600 × 300 nm.

## 2. Fundamental design of the high-precision diffractometer

The following qualifications are essential to complete the high-precision diffractometer: (i) high photon flux density of incident synchrotron radiation beam; (ii) low background intensity; (iii) easy alignment and high-performance focusing system; (iv) stable and precise axis rotation of the diffractometer.

**Figure 1:**
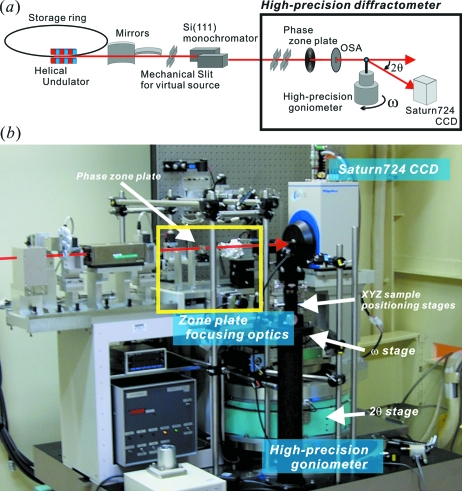
(*a*) Schematic diagram of the experimental set-up of the high-precision diffractometer system. (*b*) Photograph of the high-precision diffractometer. The red arrow and yellow box indicate the synchrotron radiation beam and the zone plate focusing optics, respectively.

Regarding qualifications (i) and (ii), we installed the diffractometer with the synchrotron radiation focusing system at the SPring-8 BL40XU helical undulator beamline (Inoue *et al.*, 2001 ▶; Kimura *et al.*, 2007 ▶). Fig. 1(*a*) ▶ shows the experimental set-up of the high-precision diffractometer system, and Fig. 1(*b*) ▶ is a photograph of the high-precision diffractometer. Fine focusing of the synchrotron radiation beam enables us to produce the incident synchrotron radiation beam with high photon flux density and small beam size, which improves the signal-to-background ratio because most of the focused synchrotron radiation beam can be incident on the sample.

For qualification (iii), we adopted a phase zone plate to focus the synchrotron radiation beam as on-axis focusing is convenient for diffraction measurements and tuning the focused beam size is easy by adjusting the longitudinal position of the phase zone plate.

Qualification (iv) is key for the structure analysis of a submicrometre-scale single powder grain. The diffraction data are measured in the oscillation mode, which is the same as in single-crystal structure analysis. In order to collect sufficiently accurate diffraction data, the submicrometre-scale single powder grain must be kept within the focused X-ray beam during the measurement. To this end, high-precision ω and 2θ rotation stages were installed in the diffractometer. To avoid eccentric rotation owing to gravitational influences on the rotation axis, vertical ω and 2θ rotation axes were adopted. The vertical rotation axis is not usually used for synchrotron radiation diffraction experiments because of horizontal polarization of the synchrotron radiation beam. However, a helical undulator, which radiates the circularly polarized synchrotron radiation beam, enabled us to use the vertical axis rotation stages.

Another advantage of the helical undulator is that the higher harmonics are emitted towards an off-axial beam direction. By extracting the central part of the radiation, therefore, the high-photon-flux beam (∼10^15^ photons s^−1^) with narrow peak-energy width (Δ*E*/*E* ≃ 2%) can be used for quasi-monochromatic synchrotron radiation X-rays. The fundamental radiation can be selected from 8 to 17 keV by changing the undulator gap. A further monochromated synchrotron radiation beam (Δ*E*/*E* ≃ 0.02%) is made using a Si(111) channel-cut monochromator.

### 2.1. Zone plate focusing optics

We designed two different phase zone plates (ZP1 and ZP2), fabricated by the NTT-AT Nanofabrication Corporation (Ozawa *et al.*, 1997 ▶). ZP1 is designed for experiments preferring higher photon energy and high photon flux density such as the structure analysis of a submicrometre-scale single powder grain. On the contrary, ZP2 is designed for experiments such as scanning X-ray microscopy requiring high spatial resolution.

ZP1 has a diameter of 100 µm, an innermost zone radius of 5.0 µm, an outermost zone width of 250 nm and a tantalum thickness of 2.5 µm. The thickness was designed to have maximum diffraction efficiency at 15 keV (∼26%). On the other hand, ZP2 has a diameter of 120 µm, an innermost zone radius of 3.0 µm, an outermost zone width of 75 nm and a tantalum thickness of 750 nm. The ideal diffraction efficiency is 12% at 8 keV. The focal lengths of ZP1 and ZP2 are 300 and 50 mm for 15 and 8 keV, respectively.

**Figure 2:**
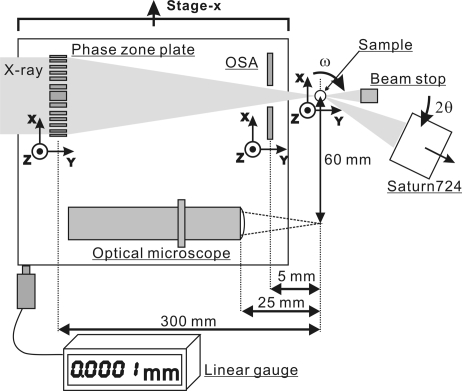
Schematic diagram of the zone plate focusing optics. The phase zone plate and OSA are aligned by the *XYZ* positioning stages. These components are mounted on the same translation stage (stage *x*), which can be moved by 60 mm on the aligned light axis to enable sample centring using the optical microscope. The position repeatability of stage *x* is monitored by the linear gauge with 100 nm resolution.

Fig. 2 ▶ shows details of the zone plate focusing optics, which has two independent *XYZ* positioning stages that align the positions of the phase zone plate and the order-sorting aperture (OSA). To align the sample onto the focused synchrotron radiation beam, an optical microscope (KEYENCE VH-Z100) with a CCD video camera can be inserted on the beam axis with a translation stage (stage *x*) on which the zone plate optics and the optical microscope are mounted in parallel. The optical microscope has a working distance of 25 mm and a magnification range from 100 to 1000, and a few micrometre-sized samples can be observed on a TV monitor. The position stability and reproducibility of the zone plate optics and the optical microscope are monitored by the linear gauge with 100 nm resolution.

### 2.2. High-precision goniometer

The high-precision goniometer has ω–2θ rotation and *XYZ* sample positioning stages (Fig. 2 ▶). Highly accurate reproducibility of the rotation centre is required for these stages to keep the micrometre-scale focused X-ray beam on the submicrometre-scale single powder grain during a measurement. We therefore adopted a high-precision air-bearing stage (Canon AB-100R) to achieve low eccentric ω-rotation within ±100 nm per 360°. The *XYZ* sample positioning stages also have 5 nm resolution.

Since the development of the sample manipulation technique of invisible submicrometre-scale powder grains has not yet been completed, several grains are presently attached to the tip of a glass fibre with a diameter of a few micrometres. The centring of the sample can be carried out systematically by using the tip of a glass fibre, not the grains, as a target. Because the diameter of the glass fibre is a few micrometres, the sample alignment can be controlled to less than 1 µm using the large magnification optical microscope inserted on the beam axis.

### 2.3. X-ray CCD detector

A high-sensitive and low-noise X-ray CCD detector (Rigaku Saturn724), having a 72 × 72 mm image area, and outputting 16-bit 2080 × 2080 pixel two-dimensional data, was used to detect weak diffraction spots of the submicrometre-scale single powder grains. The X-ray CCD is mounted on the arm of the 2θ rotation stage with a translation stage to change the camera distance from 30 to 135 mm. The 2θ resolution can be selected from 0.065° to 0.015° per pixel. The interval of the measurement owing to the dead time of the CCD camera and ω-axis positioning was about 15 s. As a result, the measurement for a data set takes less than 1 h for a micrometre-scale crystal and a few hours for a submicrometre-scale crystal. Rigaku’s *Rapid Auto System* (Rigaku, 1998 ▶) is available for processing the measured diffraction data.

## 3. Beam size and photon flux on the sample position

The synchrotron radiation beam is pre-focused by two mirrors that are installed in the optics hutch. The focal point of the mirrors is located as the virtual source at 8900 mm upstream of the phase zone plate (Fig. 1*a* ▶). The size of the virtual source can be tuned to 5 × 5 µm by the mechanical slit.

**Figure 3:**
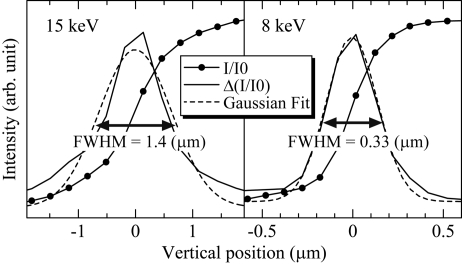
Knife-edge scan intensity data of the 15 and 8 keV microfocusing synchrotron radiation beams in the vertical direction at the focal position (solid circles). Solid and broken lines are derivatives of the measured intensity and the Gaussian fitting profiles, respectively.

Without the mechanical slit shaping of the virtual source, the photon flux densities of the 15 keV and 8 keV synchrotron radiation beams in front of the phase zone plate position were 7.9 × 10^6^ and 4.3 × 10^5^ photons s^−1^ µm^−2^, respectively. The synchrotron radiation beam is focused by the phase zone plate, and the first-order focused synchrotron radiation beam is only selected with the OSA placed before the sample position. The horizontal and vertical beam sizes at the focal position (*i.e.* the sample position) were evaluated by a knife-edge scan method using Au meshes. The beam profiles in the vertical direction at the focal position are shown in Fig. 3 ▶. Achieved performances of ZP1 and ZP2 are listed in Table 1 ▶. In the present study we achieved beam sizes of 1.4 (vertical) × 2.9 (horizontal) µm for 15 keV with ZP1 and 330 × 470 nm for 8 keV with ZP2. The photon flux densities increased to 3.1 × 10^9^ and 1.9 × 10^9^ photons s^−1^ µm^−2^. The corresponding gain factors are almost 400 and 4400, respectively. The angular divergence of the focused beam was increased to 0.019° for 15 keV with ZP1 and 0.13° for 8 keV with ZP2. Thus, in the case of ZP2, the effect of the data resolution on structure analysis should be taken into account. It should be noted that further focused beam was available by reducing the virtual source size, even though the flux becomes extremely low. The ultimate beam size of the present system was 79 × 87 nm at 8 keV with a 10 × 10 µm virtual source size. The photon flux decreased to 1.51 × 10^7^ photons s^−1^, while the photon flux density increased to 2.19 × 10^9^ photons s^−1^ µm^−2^.

## 4. Structure analysis of a submicrometre-scale BaTiO_3_ single powder grain

**Figure 4:**
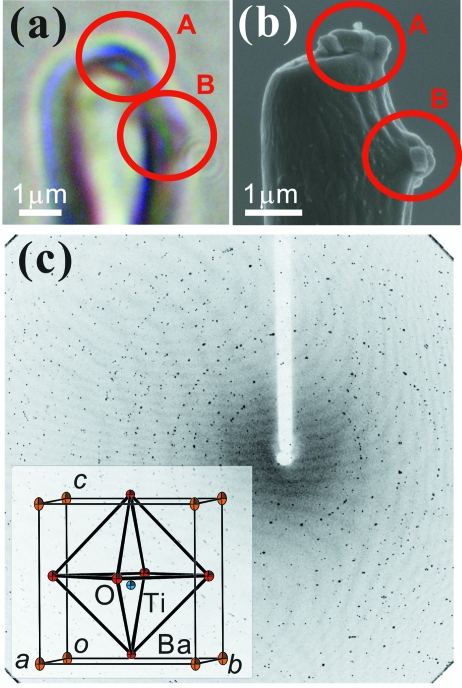
Photographs of BaTiO_3_ powder grains attached to the tip of the fine glass fibre: (*a*) optical-microscope image. (*b*) SEM image of the same sample. The size of the large grain is about 600 × 600 × 300 nm. A and B in each figure show the positions of existing BaTiO_3_ grains. (*c*) Measured diffraction image of BaTiO_3_ grains. All measured images are overlapped on one image to see Bragg diffraction spots clearly. The vertical and horizontal 2θ range of the image are ±45° and −30–60°, respectively. The inset is a 50% probability level *ORTEP* image of the refined BaTiO_3_ crystal structure.

Structure analysis was performed for a submicrometre-scale BaTiO_3_ single powder grain. The grains of BaTiO_3_ were attached to the tip of a fine glass fibre with epoxy adhesive by means of an optical microscope and a micromanipulator. Figs. 4(*a*) and 4(*b*) ▶ show the optical microscope and scanning electron microscope (SEM) images of the tip of a fine glass fibre, respectively. Several BaTiO_3_ grains were attached to the glass fibre. The maximum grain size was about 600 × 600 × 300 nm.

The diffraction data were collected in the ω-oscillation mode at room temperature. The 15 keV focused synchrotron radiation beam was prepared by ZP1. The calibration of the wavelength was carried out using the powder diffraction pattern of a NIST (National Institute of Standards and Technology) X-ray standard sample, CeO_2_ (Standard Reference Material 674a). The exposure time and the ω-axis scan step, Δω, were 15 s and 1°, respectively. With this set-up, the 360 diffraction images were collected in 180 min. The overlapped diffraction pattern of the 360 images is shown in Fig. 4(*c*) ▶. The shape of the diffraction spots does not represent significant deformation caused by the strain of the sample adhered on the glass fibre. The accidentally diffracted spots of the other BaTiO_3_ grains sometimes interfered in the diffraction pattern during ω-oscillation. These extra spots can be discriminated systematically because the diffraction pattern no longer shows the Debye–Scherrer pattern but the superposition of several single-crystal diffraction patterns. At this moment, however, it is very difficult to directly identify the sample grain corresponding to the diffraction spots among the several sample grains (Fig. 4*b* ▶).

The crystal structure was solved by direct methods (*SHELXS97*) and refined by full-matrix least-squares on *F* ^2^ (*SHELXL97*) (Sheldrick, 2008 ▶). Only the thermal vibration of the Ba atoms was refined anisotropically. The reliability factor of the refinement finally became *R*_1_ = 5.24%. The crystal structure and the determined crystallographic parameters are shown in Fig. 4(*c*) ▶ and Table 2 ▶, respectively.^1^

The crystal system of a 600 × 600 × 300 nm BaTiO_3_ single powder grain at room temperature was finally determined to be tetragonal. The values of the cell parameters, *a* = 3.9905 (13) Å and *c* = 4.0412 (14) Å, correspond to those derived from the powder diffraction studies of *a* = 3.9950 (1) Å and *c* = 4.0340 (1) Å for 400 nm-sized particles (Aoyagi *et al.*, 2002 ▶) and 3.99900 (8) Å and 4.03265 (9) Å for 430 nm-sized particles (Yashima *et al.*, 2005 ▶), respectively. This correspondence of the present result with the powder diffraction results proves that the developed diffractometry has great potential for revealing the one-on-one relationship between the crystal size and the crystal structure, if we realise the structure analysis of a single powder grain smaller than 100 nm.

## 5. Conclusion

The crystal structure of a submicrometre-scale BaTiO_3_ single powder grain was successfully determined for several single powder grains. This success proves that the diffraction measurement of a single powder grain has a sufficient advantage in the determination of unknown crystal structure for any powder-formed crystalline samples. Thus, the structure analysis of a single powder grain shall become an alternative method of powder diffraction structure analysis.

Note that the present result was obtained from one of the several grains attached on a glass fibre. Thus the manipulation technique to select only one powder grain is the remaining difficulty to be solved.

We have also developed a laser-pump and synchrotron-radiation-probe diffraction technique using the same diffractometer system (Fukuyama *et al.*, 2008*a* ▶,*b* ▶). Now we are ready to apply the structure analysis of a single powder grain for the research of the photo-induced phase transition combined with the pump and probe technique.

## Supplementary Material

Crystal structure: contains datablocks I. DOI: 10.1107/S090904950900675X/ia5038sup1.cif

Structure factors: contains datablocks I. DOI: 10.1107/S090904950900675X/ia5038sup2.hkl

## Figures and Tables

**Table 1:** Achieved performances of phase zone plates ZP1 and ZP2

	ZP1	ZP2
Photon energy (keV)	15	8
Beam size, vertical × horizontal (µm)	1.4 × 2.9	0.33 × 0.47
Photon flux (photons s^−1^)	1.25 × 10^10^	2.92 × 10^8^
Photon flux density (photons s^−1^ µm^−2^)	3.1 × 10^9^	1.9 × 10^9^
Gain factor	400	4400

**Table 2:** Crystal data and experimental details for BaTiO_3_

Formula	BaTiO_3_
Formula weight	233.24
Temperature (K)	300 (2)
Wavelength (Å)	0.83351
Crystal system	Tetragonal
Space group	*P*4*mm*
*a* (Å)	3.9905 (13)
*b* (Å)	3.9905 (13)
*c* (Å)	4.0412 (14)
Volume (Å^3^)	64.35 (4)
*Z*	1
*D*_calc_ (Mg m^−3^)	6.018
Absorption coefficient (mm^−1^)	18.037
Crystal size (nm)	600 × 600 × 300
θ range (°)	5.92–29.26
Resolution (Å)	4.04–0.85
Reflections collected/unique	468/88
*R*_int_	0.0940
Completeness to θ_max_	1.00
Data/restraints/parameters	88/0/9
*R*_1_ [*I* > 2σ(*I*)]	0.0524
*R*_*w*_ [*I* > 2σ(*I*)]	0.0994
Goodness of fit	1.168
Number of *I* > 2σ(*I*)	85
Largest diffraction peak (e Å^−3^)	2.079
Largest diffraction hole (e Å^−3^)	−1.980

## Footnotes

1 Supplementary data for this paper are available from the IUCr electronic archives (Reference: IA5038). Services for accessing these data are described at the back of the journal.

## References

[bb1] Aoyagi, S., Kuroiwa, Y., Sawada, A., Yamashita, I. & Atake, T. (2002). *J. Phys. Soc. Jpn*, **71**, 1218–1221.

[bb2] David, W. I. F. & Shankland, K. (2008). *Acta Cryst.* A**64**, 52–64. 10.1107/S0108767307064252 18156673

[bb3] David, W. I. F., Shankland, K., McCusker, L. B. & Baerlocher, Ch. (2002). Editors. *Structure Determination from Powder Diffraction Data*, pp. 1–11. Oxford University Press.

[bb4] Fukuyama, Y., Yasuda, N., Kim, J. E., Murayama, H., Ohshima, T., Tanaka, Y., Kimura, S., Kamioka, H., Moritomo, Y., Toriumi, K, Tanaka, H., Kato, K., Ishikawa, T. & Takata, M. (2008*a*). *Rev. Sci. Instrum.* **79**, 045107. 10.1063/1.2906232 18447552

[bb5] Fukuyama, Y., Yasuda, N., Kim, J. E., Murayama, H., Tanaka, Y., Kimura, S., Kato, K., Kohara, S., Moritomo, Y., Matsunaga, T., Kojima, R., Yamada, N., Tanaka, H., Ohshima, T. & Takata, M. (2008*b*). *Appl. Phys. Express*, **1**, 045001.

[bb6] Inoue, K., Oka, T., Suzuki, T., Yagi, N., Takeshita, K., Goto, S. & Ishikawa, T. (2001). *Nucl. Instrum. Methods Phys. Res. A*, **467**–**468**, 674–677.

[bb7] Kimura, S., Moritomo, Y., Tanaka, Y., Tanaka, H., Toriumi, K., Kato, K., Yasuda, N., Fukuyama, Y., Kim, J. E., Murayama, H. & Takata, M. (2007). *AIP Conf. Proc.* **879**, 1238–1241.

[bb8] Ohsumi, K., Hagiya, K. & Ohmasa, M. (1991). *J. Appl. Cryst.* **24**, 340–348.

[bb9] Ozawa, A., Tamamura, T., Ishii, T., Yoshihara, H. & Kagoshima, Y. (1997). *Microelectron. Eng.* **35**, 525–529.

[bb10] Riekel, C., Burghammer, M. & Schertler, G. (2005). *Curr. Opin. Struct. Biol.* **15**, 556–562. 10.1016/j.sbi.2005.08.013 16168633

[bb11] Rigaku Corporation (1998). *RAPID-AUTO.* Rigaku Corporation, Tokyo, Japan.

[bb12] Sheldrick, G. M. (2008). *Acta Cryst.* A**64**, 112–122. 10.1107/S0108767307043930 18156677

[bb13] Volkringer, C., Popov, D., Loiseau, T., Guillou, N., Ferey, G., Haouas, M., Taulelle, F., Mellot-Draznieks, C., Burghammer, M. & Riekel, C. (2007). *Nat. Mater.* **6**, 760–764. 10.1038/nmat1991 17873864

[bb14] Yashima, M., Hoshina, T., Ishimura, D., Kobayashi, S., Nakamura, W., Tsurumi, T. & Wada, S. (2005). *J. Appl. Phys.* **98**, 014313.

